# Interleukin-22 Deficiency Reduces Angiotensin II-Induced Aortic Dissection and Abdominal Aortic Aneurysm in ApoE-/- Mice

**DOI:** 10.1155/2022/7555492

**Published:** 2022-03-18

**Authors:** Yuan Wang, Juanjuan Li, Yulin Xu, Shichong Liao, Junlong Song, Zhiliang Xu, Wen Wei, Shan Zhu

**Affiliations:** Department of Thyroid Breast Surgery, Renmin Hospital of Wuhan University, Wuhan 430060, China

## Abstract

**Background:**

Our previous study showed that interleukin-22 (IL-22) levels were increased in patients with aortic dissection (AD). This study evaluated the effects of IL-22 on AD/abdominal aortic aneurysm (AAA) formation in angiotensin II (Ang II)-infused ApoE-/- mice.

**Methods:**

ApoE-/- mice were treated with Ang II for 28 days, and IL-22 expression was examined. In addition, the effects of IL22 deficiency on AAA/AD formation induced by Ang II infusion in ApoE-/- mice were investigated. ApoE-/-IL-22-/- mice were transplanted with bone marrow cells isolated from ApoE-/- mice or ApoE-/-IL-22-/- mice, and AAA/AD formation was observed.

**Results:**

IL-22 expression was increased in both the aortas and serum of ApoE-/- mice after Ang II infusion and was mainly derived from aortic CD4+ T lymphocytes (CD4+ TCs). IL-22 deficiency significantly reduced the AAA/AD formation as well as the maximal aortic diameter in Ang II-infused ApoE-/- mice. Decreased elastin fragmentation and reduced fibrosis were observed in the aortas of ApoE-/-IL-22-/- mice compared with ApoE-/- mice. The deletion of IL-22 also decreased aortic M1 macrophage differentiation, alleviated M1 macrophage-induced oxidative stress, and reduced aortic smooth muscle cell loss. Furthermore, M1 macrophage-induced oxidative stress was worsened and AAA/AD formation was promoted in ApoE-/-IL-22-/- mice that received transplanted bone marrow cells from ApoE-/- mice compared with those that were transplanted with bone marrow cells isolated from ApoE-/-IL-22-/- mice.

**Conclusions:**

IL-22 deficiency inhibits AAA/AD formation by inhibiting M1 macrophage-induced oxidative stress. IL-22 potentially represents a promising new target for preventing the progression of AAA/AD.

## 1. Introduction

Abdominal aortic aneurysm (AAA) is a complex clinical syndrome that frequently occurs in elderly patients and is mainly characterized by abnormal enlargement of the abdominal aortic diameter [1]. The risk of rupture increases significantly for large aortic aneurysms, and the patient dies very quickly after rupture. The current clinical treatments for large aortic aneurysms are limited to open surgery and endovascular repair; however, both strategies have remarkable limitations. Therefore, new therapeutic agents are critically needed for the prevention of AAA progression [1–3].

Interleukin-22 (IL-22) is a multifunctional cytokine in the IL-10 superfamily that is mainly derived from CD4+ T lymphocytes (CD4+ TCs) and macrophages [4, 5]. IL-22 plays a role in a variety of cardiovascular diseases through its strong inflammatory regulation ability. Neutralization of IL-22 was shown to alleviate cardiac inflammation, reduce cardiac hypertrophy, and alleviate vascular inflammation in angiotensin II- (Ang II-) treated mice, causing decreases in their blood pressure levels [6, 7]. IL-22 expression was shown to be increased in high-fat diet-fed ApoE-/- mice and to promote the development of atherosclerosis, while IL-22 deletion exerted an antiatherosclerotic effect [8, 9]. These results show that IL-22 can regulate the inflammatory response in a variety of microenvironments.

A previous study reported that IL-22 levels were significantly increased in the aortas of patients with aortic dissection (AD) and reported a further increase in IL-22 expression and substantial immune cell infiltration in the torn sections [10]. In our previous study, we found that circulating IL-22 levels were increased in patients with acute AD and positively correlated with the occurrence of AD [11]. However, the regulatory role of IL-22 in AAA/AD remains unclear. Oxidative stress has been confirmed to be involved in AAA/AD, and this study was aimed at clarifying whether IL-22 participates in AAA/AD by regulating oxidative stress.

## 2. Materials and Methods

### 2.1. Animals and Animal Models

IL-22 knockout (IL-22-/-) mice were donated by Dr. Ying Zhonglin from the People's Hospital of Guangxi Zhuang Autonomous Region, and ApoE-/- mice were purchased from the Institute of Model Zoology, Nanjing University. Both types of mice were on a C57BL/6J background. ApoE-/- and IL-22-/- mice were mated and bred, yielding ApoE-/-IL-22-/- mice. Male ApoE-/- mice and male ApoE-/-IL-22-/- mice aged 9-10 weeks were used in this experiment. The feeding conditions were the same as those described in our previous articles.

The mice were anesthetized by continuous inhalation of 1.5% isoflurane until they were immobilized. After disinfection with 75% ethanol, the neck skin was cut, and subcutaneous tissue was bluntly separated. According to the instructions provided by the manufacturer, a microosmotic pump (Alzet Model 2004) containing saline or Ang II (1000 ng/kg/min, Enzo) was implanted under the skin, and the skin was then sutured.

First, ApoE-/- mice were infused with saline or Ang II, and the IL-22 expression in each mouse was examined (*n* = 10 for each group). Second, both ApoE-/- mice and ApoE-/-IL-22 mice were treated with Ang II, and the formation of AAA and AD was investigated (*n* = 16 for each group). Finally, ApoE-/-IL-22-/- mice were transplanted with bone marrow cells isolated from ApoE-/- mice or ApoE-/-IL-22-/- mice, and AAA/AD formation was observed after Ang II infusion (*n* = 16 for each group). The mice were euthanized on day 28, after which their aortas were isolated, and the blood was collected for subsequent testing.

The use of mice and the experimental procedure were approved by the Institutional Animal Care and Use Committee of Renmin Hospital of Wuhan University (approval no. 20180915).

### 2.2. Cell Culture and Treatment

Mouse aortic vascular smooth muscle cells (SMCs), macrophages, and CD4+ TCs were used in this study. SMCs were purchased from the National Cell Collection Center of Wuhan University. Macrophages were isolated from WT mice, ApoE-/- mice, and ApoE-/-IL-22-/- mice; and CD4+ TCs were isolated from WT mice as described in our previous study [12–14]. Briefly, the mice were euthanized, and their femurs were isolated after disinfection with 75% ethanol. After removing the two ends of the femur, the bone marrow cells were flushed out with RPMI 1640 culture medium. The red blood cells were lysed and plated in a culture dish with RPMI 1640 medium containing 10% FBS. The cells were treated with 50 ng/ml macrophage colony-stimulating factor for 7 days to stimulate macrophage differentiation, and bone marrow-derived macrophages were then obtained. The spleens of the euthanized mice were isolated, and single-cell suspensions were prepared. After the red blood cells were lysed, the lymphocytes were separated by a lymphocyte separator, and CD4+ TCs were positively selected using an autoMACS separator and CD4 magnetic beads (Miltenyi) and then activated by a cell stimulation cocktail (2 *μ*L/mL, eBioscience).

In part I of the experiment, both CD4+ TCs and macrophages were treated with Ang II (100 ng/ml) or saline for 24 hours, and the IL-22 mRNA levels in each group were detected.

In part II, ApoE-/- and ApoE-/-IL-22-/- macrophages were treated with Ang II, and the inducible nitric oxide synthase (iNOS) intensity, M1 macrophage-related marker mRNA levels, and oxidative stress were then measured.

In part III, SMCs were treated with the supernatant collected from part II, and some of the cells were treated with SOD (100 U) or Ang II [15]. Then, the mRNA expression levels of Bax and Bcl2 in SMCs were measured.

### 2.3. Bone Marrow Transplantation

Bone marrow transplantation was performed as described in a previous study [16, 17]. Briefly, bone marrow was isolated as described above, and the bone marrow cells were isolated by density gradient separation. Recipient ApoE-/-IL-22-/- mice were irradiated with a lethal dose of 9.5 Gy. After 24 hours, the mice were divided into 2 groups and received an intravenous injection of 3 × 10^6^ bone marrow cells isolated from ApoE-/- mice or ApoE-/-IL-22-/- mice. The microosmotic pumps were implanted at 6 weeks after bone marrow transplantation.

### 2.4. Aortic Ultrasound and AAA Definition

The abdominal hair was removed with depilatory cream, and the skin was cleaned with wet gauze. After anesthetization as previously described, the mice were placed in a supine position on the heating plate. After evenly applying the heated couplant to the mouse abdomen, and the ultrasonic probe was placed close to the abdomen to collect visual images of the abdominal aorta and thereby calculate the maximum inner diameter of the abdominal aorta using an imaging system (Vevo 1100, VisualSonics).

In this study, ApoE-/- mice infused with saline were used as a control. The aortic ultrasound results showed that the average maximum aortic diameter of these mice was 0.94 mm. According to the literature, AAA is defined as the aortic diameter being increased by 50% compared with the normal diameter. Therefore, AAA was herein defined as an aortic diameter exceeding 1.43 mm.

### 2.5. Histological Analysis

After aortic rings were isolated and cut, the aortas were immediately fixed in 4% paraformaldehyde. After dehydration and paraffin embedding, the aortic rings were cut into 4 to 5 *μ*m thick sections and mounted onto slides. Hematoxylin and eosin (H&E) staining was used to analyze aortic wall thickness, Masson staining was used to detect aortic fibrosis, and elastic van Gieson (EVG) staining was performed to detect aortic fibrin rupture. These general methodologies are provided in the Supplementary Material. Mouse anti-IL-22, mouse anti-iNOS, and mouse anti-*α*-smooth muscle actin (*α*-SMA) antibodies (all from GeneTex) were used for the immunofluorescence staining of the aortic markers IL-22, iNOS, and SMA; and mouse anti-F4/80 and anti-IL-22 antibodies were used to determine whether IL-22 was derived from aortic macrophages by immunofluorescence double staining. In addition, aortic apoptotic cells were labeled using a TUNEL assay kit (Millipore) according to the manufacturer's instructions. The statistical analyses of the EVG and immunofluorescence staining results were described in a previous study [18]. Briefly, the elastin degradation score was defined as follows: grade 1, no degradation; grade 2, mild elastin degradation; grade 3, severe elastin degradation; and grade 4, aortic rupture. The immunofluorescence results were quantified by 3 observers blinded to the sample identities using an arbitrary grading system to estimate the degree of positive staining for each marker as follows: score 1: 0-25% positive staining in medium; score 2: 26-50% positive staining in medium; score 3: 51-75% positive staining in medium; and score 4: 76-100% positive staining in medium.

### 2.6. Flow Cytometry

After the aorta was separated, the surrounding adipose tissue was removed and cut into small pieces with fine scissors, digested with phosphate-buffered saline (PBS) containing collagenase IX (125 U/ml), collagenase I (450 U/ml), hyaluronidase I (60 U/ml), calcium, and magnesium at 37°C for 1 hour and stirred continuously. After centrifugation, the supernatant was removed, and the cells were collected and resuspended in a 100 *μ*l of FACS buffer (BD sciences). Subsequently, fluorescein isothiocyanate (FITC) mouse anti-F4/80 (0.5 *μ*l/test, BD sciences) and phycoerythrin-labeled (PE) mouse anti-CD86 (0.5 *μ*l/test, BD sciences) antibodies were added and incubated at room temperature in the dark for 45 min. Then, the cells were collected and fixed in 4% neutral paraformaldehyde for subsequent analysis with the FlowJo 10 software.

### 2.7. Western Blot and RT–qPCR Analyses

Western blot and quantitative RT–qPCR analyses were performed according to our previous studies [12–14]. In this study, the aortic expression levels of IL-22, Bax, Bcl2, and GAPDH (all from GeneTex) were determined by western blot analysis, and the mRNA levels of IL-22, iNOS, CD38, CD80, and CD86 in macrophages and of Bax and Bcl2 were measured by RT–qPCR. The primers are provided in Supplementary Material Table S1.

### 2.8. Detection of Serum IL-22 and Aortic Oxidative Stress

Serum was collected from each blood sample after centrifugation at 800 × g for 15 min. According to the pretest results, the serum was diluted 4-fold, and the IL-22 levels in each serum sample were then examined using mouse the IL-22 ELISA kits (Thermo Fisher Scientific) according to the manufacturer's instructions.

In addition, aortas were lysed with RIPA lysis buffer, and the supernatants were collected and quantified. Then, the malondialdehyde (MDA) level, glutathione (GSH) level, and SOD activity in each sample were measured using the appropriate kits (all from Beyotime Biotechnology) according to the manufacturers' instructions.

### 2.9. Statistical Analysis

All data are presented as the mean ± standard deviation and were analyzed by the GraphPad Prism 8 software. Differences in the means between the 2 groups were determined by unpaired 2-tailed Student's *t*-test, and differences in the means among multiple groups were determined by ANOVA followed by Tukey's multiple comparison and Bonferroni post hoc tests. Fisher's exact test was performed to compare AAA/AD incidence rates, and the log-rank test was used to analyze the survival curve data. In this study, differences were considered statistically significant for *p* values less than 0.05.

## 3. Results

### 3.1. Ang II Infusion Increased IL-22 Expression in ApoE-/- Mice

**Figure 1:**
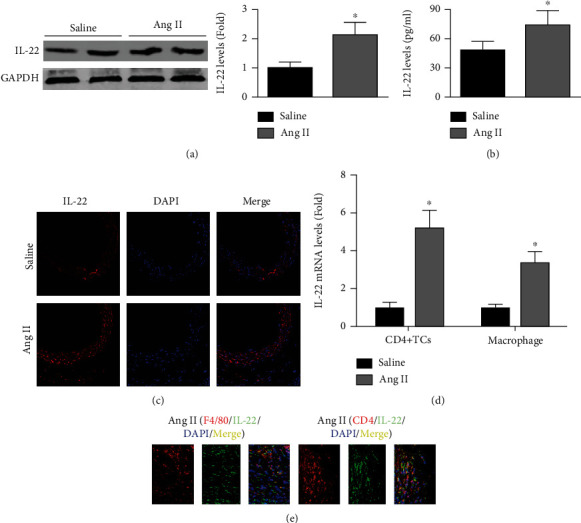
Effects of Ang II infusion for 4 weeks on IL-22 expression. (a and b). The aortic and serum IL-22 levels in the saline-infused group and Ang II-infused group were examined by western blotting and ELISA. (c). Aortic IL-22 expression was assessed by immunofluorescence analysis (200x). (d). Effect of Ang II treatment on the IL-22 mRNA expression in macrophages and CD4+ T lymphocytes. (e). Double staining with anti-F4/80 and anti-IL-22 antibodies and anti-CD4 and anti-IL-22 antibodies in Ang II-infused mice (200x). *N* = 5 − 8 in each group. ^∗^*p* < 0.05 vs. the saline group.

The western blot and immunofluorescence staining results showed that Ang II infusion for 4 weeks increased aortic IL-22 expression in ApoE-/- mice (Figures 1(a) and 1(c)). Ang II also elevated serum IL-22 levels (Figure 1(b)). Treatment with Ang II increased the IL-22 mRNA expression by 4.2-fold in CD4+ TCs and by 2.4-fold in macrophages (Figure 1(d)). Double staining with anti-F4/80 and anti-IL-22 antibodies showed that aortic macrophages were the source of IL-22 (Figure 1(e)).

### 3.2. IL-22 Deletion Prevented Ang II-Induced AAA/AD Formation in ApoE-/- Mice

**Figure 2:**
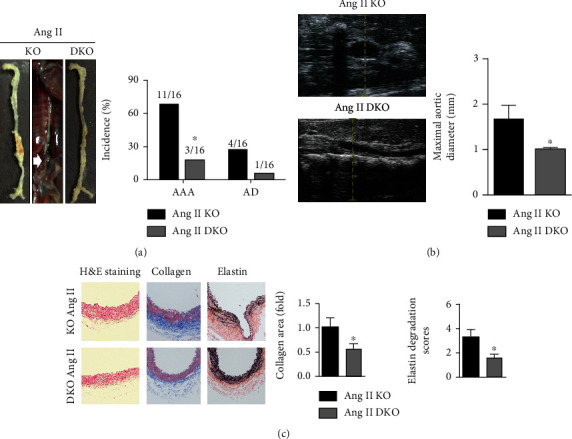
Effects of IL-22 deficiency on AAA/AD formation in Ang II-infused ApoE-/- mice. (a) The incidence of AAA/AD in the two groups. (b) The maximum aortic diameter was measured by abdominal aorta ultrasound. (c) H&E staining, Masson staining, and EVG staining were performed, and collagen expression and elastin fragmentation were analyzed (200x). *N* = 6 − 8 in each group. KO indicates ApoE-/- mice and DKO indicates ApoE-/- mice. ^∗^*p* < 0.05 vs. the Ang II-KO group.

No significant differences in baseline systolic blood pressure levels were observed between ApoE-/- mice and ApoE-/-IL-22-/- mice, while ApoE-/-IL-22-/- mice exhibited lower systolic blood pressure after Ang II infusion (Supplementary material, Figure 1). During Ang II infusion, AD was observed in 4 mice from the ApoE-/- group and in 1 mouse from the ApoE-/-IL-22-/- group (4/16 vs. 1/16, Figure 2(a)). IL-22 deletion also significantly decreased the AAA incidence and the maximum aortic diameter induced by Ang II in ApoE-/- mice (Figures 2(a) and 2(b)). In addition, when compared with Ang II-infused ApoE-/- mice, Ang II-infused ApoE-/-IL-22-/- mice showed reduced marginal adventitial thickening, decreased collagen deposition in the adventitia of the aorta, and reduced elastin fragmentation (Figure 2(c)).

### 3.3. IL-22 Knockout Alleviated M1 Macrophage-Induced Aortic Oxidative Stress in Ang II-Infused ApoE-/- Mice

**Figure 3:**
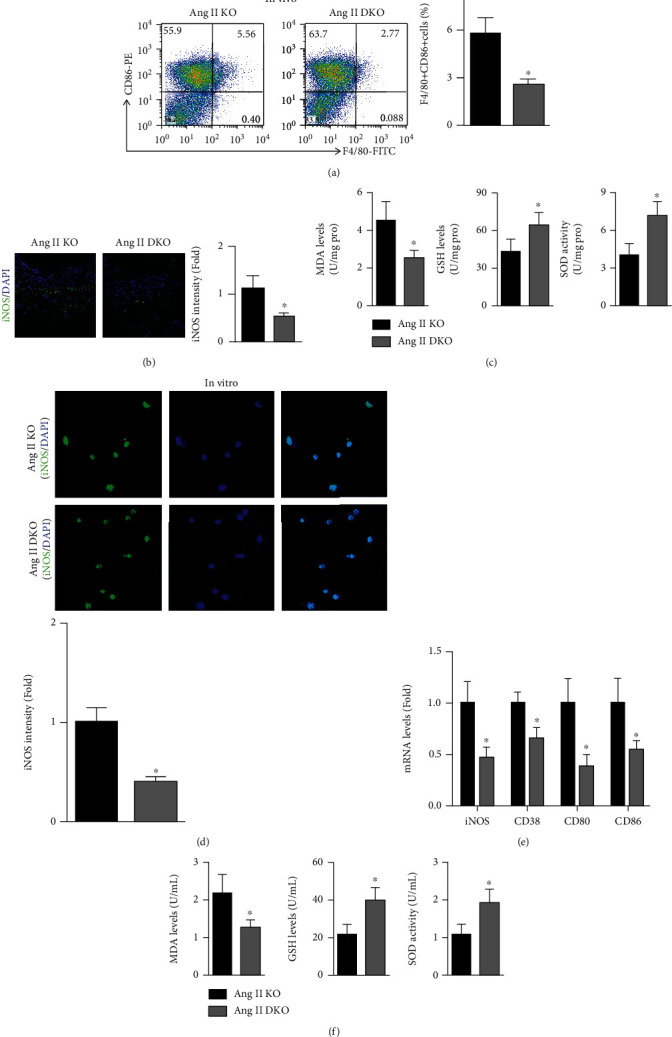
Effects of IL-22 deletion on M1 macrophage-induced oxidative stress in Ang II-treated ApoE-/- mice. (a) The levels of F4/80 + CD86+ cells in these two groups were measured by flow cytometry. (b) Aortic iNOS intensity in each group (200x). (c) The MDA levels, GSH levels, and SOD activity were analyzed. (d) The iNOS intensity in macrophages was examined (200x). (e) The mRNA levels of iNOS, CD38, CD80, and CD86 in vitro were measured. (f) The MDA levels, GSH levels, and SOD activity in the supernatant were examined. *N* = 6 − 8 in each group. KO indicates ApoE-/- mice and DKO indicates ApoE-/- mice. ^∗^*p* < 0.05 vs. the Ang II-KO group.

M1 macrophage differentiation was examined by flow cytometry, and the results showed that IL-22 knockout decreased the F4/80 + CD86+ cell levels in Ang II-infused ApoE-/- mice (Figure 3(a)). In addition, the Ang II-induced fluorescence intensity of aortic iNOS in ApoE-/- mice was decreased significantly by IL-22 knockout (Figure 3(b)). Furthermore, IL-22 deletion decreased the MDA level, increased the GSH level, and increased the SOD activity (Figure 3(c)). In vitro, IL-22 deficiency also decreased the iNOS intensity, reduced the mRNA expression of M1 macrophage-related markers, including iNOS, CD38, CD80, and CD86, and alleviated oxidative stress factors in the supernatants of Ang II-treated ApoE-/- macrophages (Figures 3(d)–3(f)).

### 3.4. IL-22 Deletion Decreased Ang II-Induced SMC Loss in ApoE-/- Mice

**Figure 4:**
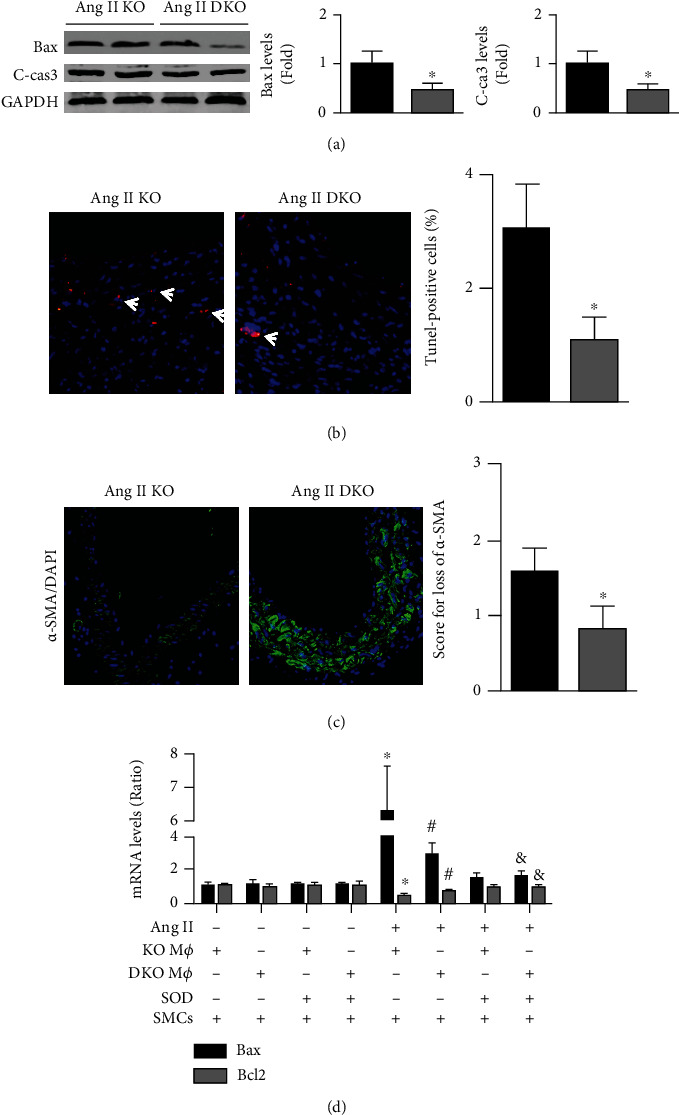
Effects of IL-22 knockout on Ang II-induced SMCs loss in ApoE-/- mice. (a) Aortic Bax and Bcl2 expression was quantified and analyzed; ^∗^*p* < 0.05 vs. the Ang II-KO group. (b) TUNEL-positive cells are marked; ^∗^*p* < 0.05 vs. the Ang II-KO group (200x). (c) The levels of aortic SMCs were examined; ^∗^*p* < 0.05 vs. the Ang II-KO group (200x). (d) Bax and Bcl2 mRNA levels in SMCs in each group; ^∗^*p* < 0.05 vs. the KO Mø + SMCs group, ^#^*p* < 0.05 vs. the Ang II + KO Mø + SMCs group, and ^&^*p* < 0.05 vs. the Ang II + DKO Mø + SMCs group. *N* = 6 − 8 in each group. KO indicates ApoE-/- mice and DKO indicates ApoE-/-IL-22-/- mice.

The western blot results showed that IL-22 deletion decreased the aortic levels of Bax and cleaved caspase-3 in Ang II-infused ApoE-/- mice (Figure 4(a)). Fewer TUNEL-positive cells and lower *α*-SMA loss scores were observed in the ApoE-/-IL-22-/- group compared with the ApoE-/- group (Figures 4(b) and 4(c)). In vitro, application of the supernatant of Ang II-treated ApoE-/- macrophages significantly increased the expression of Bax and decreased the expression of Bcl-2, while application of the supernatant of Ang II-treated ApoE-/-IL-22-/- macrophages decreased the Bax mRNA levels and increased those of Bcl2; no differences were observed in the Bax and Bcl2 mRNA levels in SMCs after the addition of SOD (Figure 4(d)).

### 3.5. Transplanted Bone Marrow Cells Isolated from ApoE-/- Mice Promoted AAA/AD Formation in Ang II-Infused ApoE-/-IL-22-/- Mice

**Figure 5:**
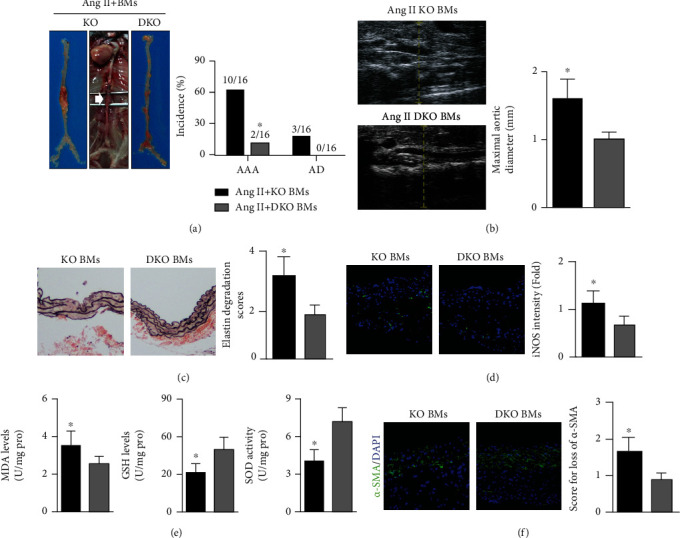
Effect of bone marrow transplantation on AAA/AD formation in Ang II-treated ApoE-/-IL-22-/- mice. (a and b) The AAA/AD incidence and maximum aortic diameters in the two groups. (c) EVG staining was performed, and elastin fragmentation was analyzed (200x). (d) The aortic iNOS intensity was examined in the different groups (200x). (e) The aortic MDA levels, GSH levels, and SOD activity were analyzed. (f) SMCs loss in the two groups. *N* = 5 − 8 in each group. KO BMs indicate transplanted bone marrow cells isolated from ApoE-/- mice, and DKO BMs indicate transplanted bone marrow cells isolated from ApoE-/-IL-22-/- mice.

ApoE-/-IL-22-/- mice transplanted with bone marrow cells isolated from ApoE-/- mice exhibited increased aortic IL-22 expression compared with that in mice that were transplanted with bone marrow cells isolated from ApoE-/-IL-22-/- mice (Supplementary Material, Figure 2). In Ang II-infused ApoE-/-IL-22-/- mice, AD occurred in 3 mice after ApoE-/- bone marrow cell transplantation and in 2 mice after ApoE-/-IL-22-/- bone marrow cell transplantation (Figure 5(a)). The transplantation of bone marrow cells from ApoE-/- mice promoted AAA formation, increased the maximum aortic diameter, and promoted elastin fragmentation (Figures 5(a)–5(c)). In addition, stronger iNOS fluorescence intensity, higher aortic oxidative stress levels, and more SMC loss were observed in ApoE-/-IL-22-/- mice transplanted with bone marrow cells from ApoE-/- mice compared with those of the other groups (Figures 5(d)–5(f)).

## 4. Discussion

AAA is an invisible killer that seriously threatens the health of humans, and preventing AAA progression and rupture is very important for patients, especially elderly patients. Previous studies have focused on the local inflammatory response in the aorta, which is an important factor underlying the progression of AAA and directly affects patient prognosis. In the present study, we found that IL-22 deletion reduced aortic M1 macrophage differentiation, decreased oxidative stress and SMC loss, and prevented AAA/AD formation in Ang II-infused ApoE-/- mice. In addition, ApoE-/-IL-22-/- mice that were transplanted with bone marrow cells from ApoE-/- mice exhibited exacerbated SMC loss and increased AAA/AD formation. These results suggest that IL-22 is involved in the progression of AAA/AD by regulating M1 macrophage-induced oxidative stress, making it a potential clinical target for the treatment and prevention of AAA and AD.

Bone marrow cells are one of the most important sources of immune cells in the body. When stimulated by physiological or external pathological factors, bone marrow cells can differentiate into other types of immune cells, including monocytes, macrophages, and dendritic cells, and exert anti-inflammatory or proinflammatory effects [19, 20]. Among them, monocytes exhibit a classic anti-inflammatory phenotype and an intermediate proinflammatory phenotype typical of amorphous monocytes [21]. Macrophages can be divided into proinflammatory M1 macrophages and anti-inflammatory M2 macrophages [22]. Tolerant dendritic cells play an anti-inflammatory role, while mature dendritic cells play a proinflammatory role [23]. Numerous studies have confirmed that the infiltration of immune cells, including monocytes and macrophages, in the aortic wall is increased, and AAA progression is significantly inhibited when immune cell infiltration is reduced [24]. In addition, the infiltration of macrophages and CD4+ TCs into the torn section is further increased in the aortic vascular tissues of AD patients [10]. Considering that AAA and AD have the same pathogenesis, the infiltration and differentiation of immune cells is thought to not only affect the progression of AAA but also to be potentially related to AAA rupture.

Macrophages are the most studied immune cells in AAA/AD and have been reported to be involved in the AAA/AD process. Ye et al. found that the aortic levels of M1 macrophages were immediately increased after Ang II administration, peaked in the second week, and lasted until the end of the fourth week, while the expression of Arg-1 decreased significantly in the first week, increased in the second week, and lasted until the fourth week [14]. In animal studies, abdominal aortic diameter enlargement and AAA formation were observed in fewer than 14 days after Ang II infusion, and some mice even died due to AD [25, 26]. This evidence suggests that M1 macrophages but not M2 macrophages play a leading role in the occurrence of AD and the formation of AAA/AD. In addition, previous studies have shown that IL-22 directly exerts biological effects on nonimmune cells, and an increasing number of studies have shown that IL-22 can also regulate macrophage differentiation and participate in various disease processes [27, 28].

In this study, ApoE-/- mice were infused with Ang II to establish an animal model of AAA/AD, and IL-22 deficiency significantly reduced the incidence of AAA/AD. These data confirm that IL-22 knockout has anti-AAA and anti-AD effects. To clarify the mechanisms by which IL-22 regulates AAA/AD formation, the levels of M1 macrophages in the aortic wall were examined. The flow cytometry results showed that the percentages of F4/80 + CD86+ cells were significantly reduced by IL-22 deletion, and immunofluorescence staining showed lower fluorescence intensity of iNOS in the aortas of ApoE-/-IL-22-/- mice than in those of ApoE-/- mice. Similar effects of IL-22 deletion on Ang II-induced ApoE-/- macrophages were observed in vitro. These data suggest that IL-22 is involved in the process of AAA/AD by regulating M1 macrophage differentiation. To test this hypothesis, ApoE-/-IL-22-/- mice were transplanted with bone marrow cells derived from ApoE-/- mice or ApoE-/-IL-22-/- mice. The results showed that the transplantation of bone marrow cells derived from ApoE-/- mice increased aortic IL-22 expression, increased M1 macrophage differentiation, and promoted the occurrence of AAA/AD. These results demonstrated that IL-22 is critical for the Ang II-induced differentiation of M1 macrophages and that IL-22 regulates the AAA/AD process by promoting the differentiation of M1 macrophages in the aorta. Elevated blood pressure was demonstrated to be associated with the occurrence of AAA/AD [29]. To exclude the influence of blood pressure, the effects of IL-22 on Ang II-induced M1 macrophage differentiation were examined in vitro. The results showed that IL-22 knockout inhibited M1 macrophage differentiation which suggests that IL-22 knockout may reduced AAA/AD incidence when the effect of blood pressure was excluded.

When exploring the mechanism of AAA, researchers mostly focused on M1 macrophages which can mediate inflammatory response, suggesting that M1 macrophages play a leading role in the process of AAA. Our previous studies found that M1 macrophages were at a high level after Ang II perfusion, while M2 macrophages decreased in the early stage and increased feedback in the middle and late stage [14]. AAA can be formed in the early-middle stage after Ang II infusion, while more collagen fiber deposition can be observed in the aortic wall of AAA, and the collagen deposition of vessels with AAA is more serious [25]. Considering that M2 macrophages are the main source of collagen fibers and play a role in tissue repair, suggests that collagen is the repair performance after vascular injury caused by M1 macrophages in AAA, that is, decrease collagen means lighter vascular injury. Just like myocardial fibrosis in the infarct site after a myocardial infarction. Additionally, collagen can prevent excessive expansion of vascular wall and is beneficial to maintaining the structure of blood vessels. While reducing collagen that maintain damaged blood vessels may make vascular expansion unrestricted in AAA, which may be harmful. At present, there are few relevant studies, and more studies are needed to confirm it.

Vascular SMCs are the most important component of the aorta and are essential for maintaining its normal structure and function. Data from clinical studies and animal experiments indicate that excessive apoptosis and SMC loss occur in aortic aneurysms and AD, while a reduction in SMC apoptosis significantly inhibits the rupture of aortic aneurysms, which is associated with death [29–31]. These studies suggested that the vascular function and structural damage caused by excessive vascular SMC apoptosis are critical for the occurrence and development of aortic aneurysms and AD.

As the tolerance of SMCs to excessive oxidative stress is weakened, the enhancement of aortic vascular oxidative stress is one of the most important factors leading to excessive SMC apoptosis [32, 33]. In addition, M1 macrophages are an important source of oxidative stress and have been shown to be associated with AAA formation [33, 34]. To further clarify the mechanism by which IL-22 regulates AAA/AD progression, several oxidative stress markers were examined, and the results showed that IL-22-/- deletion significantly reduced the MDA levels and increased the GSH levels and SOD activity in aortas and in vitro, suggesting that IL-22 knockout reduces aortic oxidative stress. In addition, SMCs were treated with the supernatant from Ang II-treated macrophages, and treatment with the supernatant of ApoE-/-IL-22-/- macrophages significantly reduced SMC apoptosis compared with that resulting from treatment with the ApoE-/- macrophage supernatant. In addition, ApoE-/-IL-22-/- mice that were transplanted with bone marrow cells derived from ApoE-/- mice exhibited aggravated aortic oxidative stress. These results suggest that IL-22 is involved in the progression of AAA/AD by regulating M1 macrophage-induced aortic oxidative stress.

In conclusion, our study demonstrates that IL-22 is involved in the progression of AAA by regulating macrophage differentiation-related aortic oxidative stress and that IL-22 has potential as a clinical target for the prevention of AAA and AD. However, it is unclear what mediators induced by M1 macrophages led to SMC apoptosis, and further research is necessary.

## Data Availability

We declare that the materials described in the manuscript, including all relevant raw data, will be freely available to any scientist wishing to use them for noncommercial purposes, without breaching participant confidentiality.

## Supplementary Materials

Supplementary Materials General steps for H&E staining. General steps for Masson staining. General steps for EVG staining. Figure S1: systolic blood pressure as measured at different time points via the tail-cuff method. Figure S2: aortic IL-22 levels in the two groups. Table S1: primers used in this study.
