# Supplementary material for: Interleukin-22 Deficiency Reduces Angiotensin II-Induced Aortic Dissection and Abdominal Aortic Aneurysm in ApoE-/- Mice
Source: Oxid Med Cell Longev. 2022 Mar 18;2022:7555492. doi: 10.1155/2022/7555492 (PMC8956387; doi:10.1155/2022/7555492)
Supplement: Supplementary Materials — General steps for H&E staining. General steps for Masson staining. General steps for EVG staining. Figure S1: systolic blood pressure as measured at different time points via the tail-cuff method. Figure S2: aortic IL-22 levels in the two groups. Table S1: primers used in this study. [file 7555492.f1.doc]

**Supplementary Material**

**General steps for H&E staining**

1. The slices were sequentially incubated with xylene I (20 min), xylene II (20 min) anhydrous ethanol I (5 min), anhydrous ethanol II (5 min), and 75% alcohol (5 min) and then washed with tap water.

2. The slices were incubated with hematoxylin dye (3-5 min), washed with tap water, differentiated with differentiation solution, washed with tap water, returned to blue by staining with hematoxylin-eosin, and rinsed with running water.

3. The slices were dehydrated sequentially with 85% alcohol (5 min) and 95% alcohol (5 min) before being dyed with eosin (5 min).

4. The slices were sequentially incubated with anhydrous ethanol I (5 min), anhydrous ethanol II (5 min), anhydrous ethanol III (5 min), xylene I (5 min) and xylene II (5 min) before being sealed with neutral gum.

5. The slices were examined under a microscope, and images were acquired and analyzed.

**General steps for Masson staining**

1. The slices were sequentially incubated with xylene I (20 min), xylene II (20 min), anhydrous ethanol I (5 min), anhydrous ethanol II (5 min), and 75% alcohol (5 min) before being washed with tap water.

2. The slices were soaked in Masson A solution overnight and washed with tap water.

3. The slices were soaked in a equal mixture of Masson solution B and Masson solution C for 1 min, washed with tap water, differentiated with 1% hydrochloric acid alcohol, and washed with tap water.

4. The slices were then soaked in Masson D solution for 6 min and rinsed with tap water.

5. The cells were soaked in Masson E solution for 1 min.

6. Without washing with water, the solution was drained slightly, and the slices were directed dyed with Masson F solution for 20-30 s.

7. The sections were rinsed and differentiated with 1% glacial acetic acid and then dehydrated with two cylinders of anhydrous ethanol.

8. The slices were sequentially incubated with a third cylinder of anhydrous ethanol (5 min) and xylene (5 min) to induce transparency and were then sealed with neutral gum.

9. The slices were examined under a microscope, and images were acquired and analyzed.

**General steps for EVG staining**

1. The slices were sequentially incubated with xylene I (20 min), xylene II (20 min), anhydrous ethanol I (5 min), anhydrous ethanol II (5 min), and 75% alcohol (5 min) before being washed with tap water.

2. The EVG solution was prepared two days in advance (EVG dye A: EVG dye B: EVG dye C=5:2:2), and the slices were incubated with the EVG solution (5 min) and then rinsed with running water.

3. EVG dye B was diluted twice and slightly differentiated, and the slices were then washed with tap water. These protocols were then repeated under the microscope to control the degree of differentiation until the elastic fiber became purple and black and the background was gray and white (nearly colorless).

4. The VG dye solution (EVG dye D: EVG dye E=1:9) was prepared and then incubated with the slices (1-3 min, the dyeing time depended on the elastic fiber composition in the tissue; the collagen appeared as a light color if the dyeing time was too short, and the elastic fibers faded if the dyeing time was too long). The slices were then washed with water quickly and dehydrated with three cylinders of anhydrous ethanol.

5. The samples were incubated with two cylinders of clean xylene to induce transparency (20 s and 5 min, xylene was dedicated and not shared with other xylenes), followed by sealing with neutral rubber.

6. The slices were examined under a microscope, and images were acquired and analyzed.


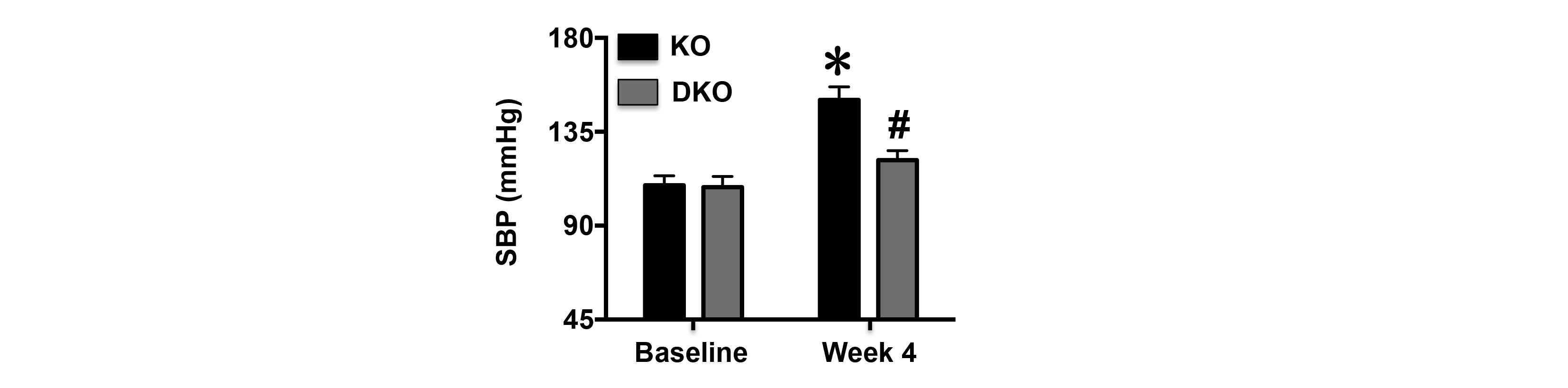


**Figure S1:** Systolic blood pressure as measured at different time points via the tail cuff method.


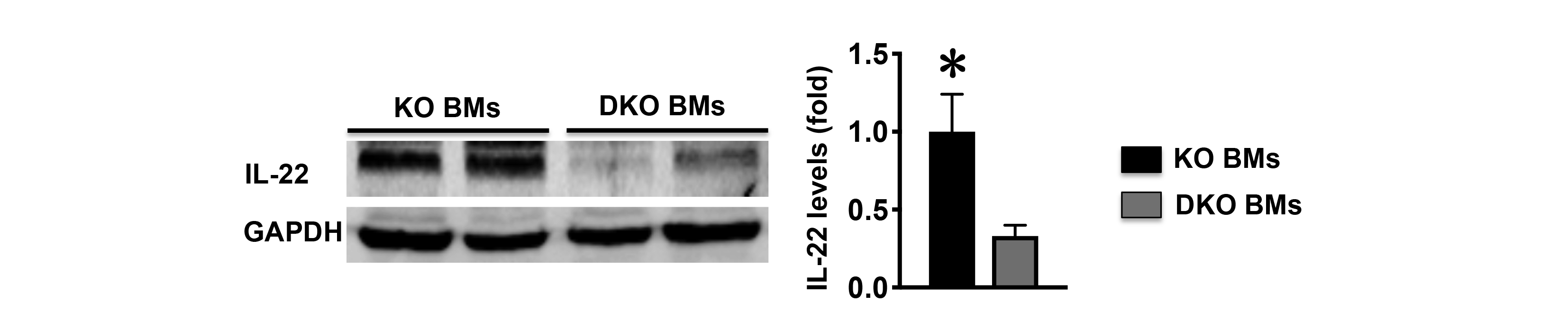


**Figure S2:** Aortic IL-22 levels in the two groups.

Table S1: Primers used in this study.

| **Gene** | **Forward primer** | **Reversed primer** |
| --- | --- | --- |
| IL-22 | CAACTTCCAGCAGCCATACA | GTTGAGCACCTGCTTCATCA |
| iNOS | CGAAACGCTTTCACTTCCAA | TGAGCCTATATTGCTGTGGCT |
| CD38 | TCTCTAGGAAAGCCCAGATCG | GTCCACACCAGGAGTGAGC |
| CD80 | GGCCTGAAGAAGCATTAGCTG | GAGGCTTCACCTAGAGAACCG |
| CD86 | GAGGCTTCACCTAGAGAACCG | TGTCAGCGTTACTATCCCGC |
| Bax | TGAGCGAGTGTCTCCGGCGAAT | GCACTTTAGTGCACAGGGCCTTG |
| Bcl2 | TGGTGGACAACATCGCCCTGTG | GGTCGCATGCTGGGGCCATATA |
| GAPDH | AACTTTGGCATTGTGGAAGG | CACATTGGGGGTAGGAACAC |
